# Clinical Outcomes of Colorectal Endoscopic Full‐Thickness Resection Using the Full‐Thickness Resection Device: A Multicenter Study

**DOI:** 10.1002/deo2.70365

**Published:** 2026-07-03

**Authors:** Vicki McGarrigle, Yuto Shimamura, Leonardo Zorron Cheng Tao Pu, Marios Efthymiou, Rhys Vaughan, Cameron Schauer, Anurag Sekra, Erin Horsfall, Alexandra Simpson, Rajvinder Singh, Nicholas Wan, Milan Bassan, Martin Harb, Ben Terkasher, Frank Weilert, Saurabh Gupta, Kwang Chien Yee, Rajan Patel, Ibrahim Hassan, Byron Theron, Sujievvan Chandran

**Affiliations:** ^1^ Department of Gastroenterology and Hepatology Austin Health Melbourne Australia; ^2^ Department of Gastroenterology Te Whatu Ora – Health NZ, Waitemata Auckland New Zealand; ^3^ Department of Gastroenterology Te Whatu Ora – Health NZ Counties Manukau Auckland New Zealand; ^4^ Department of Gastroenterology Lyell McEwin Hospital Elizabeth Vale South Australia Australia; ^5^ Faculty of Health and Medical Sciences The University of Adelaide Adelaide South Australia Australia; ^6^ Gastroenterology and Hepatology Department Liverpool Hospital Liverpool New South Wales Australia; ^7^ South West Sydney Clinical Campuses University of NSW Sydney Sydney New South Wales Australia; ^8^ Department of Gastroenterology St George Private Hospital Kogarah New South Wales Australia; ^9^ Department of Gastroenterology Te Whatu Ora – Health NZ Waikato District Health Board Hamilton New Zealand; ^10^ Department of Gastroenterology Sydney Adventist Hospital Sydney New South Wales Australia; ^11^ Department of Gastroenterology Calvary Lenah Valley Hospital Endoscopy Unit Lenah Valley Tasmania Australia; ^12^ Department of Gastroenterology Te Whatu Ora – Health NZ Christchurch Hospital Christchurch New Zealand; ^13^ Department of Gastroenterology and Hepatology Te Whatu Ora – Health NZ, Te Toka Tumai Auckland City Hospital Auckland New Zealand; ^14^ Department of Gastroenterology Te Whatu Ora – Health NZ Te Tai Tokerau Northland District Health Board Whangārei New Zealand

**Keywords:** colonoscopy, endoscopic full‐thickness resection, gastrointestinal endoscopy, postoperative outcomes, therapeutic endoscopy

## Abstract

**Background:**

Endoscopic full‐thickness resection (EFTR) using the full‐thickness resection device (FTRD) is an emerging technique for the management of complex colorectal lesions and early colorectal cancers. Although EFTR offers a minimally invasive alternative to surgery, data on efficacy, safety, and predictors of adverse events (AEs) remain limited. This study evaluated outcomes of colorectal EFTR using FTRD and identified patient‐ and lesion‐related factors associated with AEs.

**Methods:**

This multicenter retrospective study included patients who underwent colorectal EFTR with FTRD across 13 centers in Australia and New Zealand. Patient demographics, lesion characteristics, procedural outcomes, R0 resection rates, and AEs were analyzed.

**Results:**

A total of 170 EFTR procedures were performed. The mean patient age was 67 years (standard deviation [SD] 13.7), and 57% were male. Mean procedure time was 51 min (SD 28.5). The most common indication was non‐lifting lesions (36%). Technical success was achieved in 89% of cases. Adenocarcinoma was confirmed on histopathology in 23% of lesions, with an R0 resection rate of 79%. Recurrence occurred in 7.4% of cases during a median follow‐up of 5 months (interquartile range [IQR] 8.5). Early and delayed AEs occurred in 4.1% and 12.9%, respectively, including post‐procedural bleeding (*n *= 11), perforation (*n *= 5), and post‐polypectomy syndrome (*n* = 2). Among 41 procedures involving appendiceal orifice lesions, appendicitis developed in 17% (*n* = 7). AEs were significantly associated with female sex (64% vs. 40%, *p* = 0.03) and appendiceal orifice lesions (46% vs. 21%, *p *= 0.02).

**Conclusion:**

Colorectal EFTR using FTRD is effective for the resection of complex colorectal lesions. Careful patient selection and risk stratification are essential to optimize outcomes.

**Abbreviations**
AE: adverse event
EFTR: endoscopic full‐thickness resection
EMR: endoscopic mucosal resection
ESD: endoscopic submucosal dissection
FTRD: full‐thickness resection device
IQR: interquartile range
SD: standard deviation

## 1 Introduction

**FIGURE 1:**
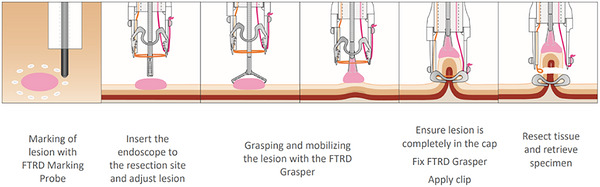
Schematic diagram of endoscopic full‐thickness resection using the full‐thickness resection device (FTRD). Schematic illustration of endoscopic full‐thickness resection performed using the FTRD (Ovesco Endoscopy, Tübingen, Germany), demonstrating lesion capture, clip deployment, and full‐thickness resection. Images provided by Ovesco Endoscopy.

**FIGURE 2:**
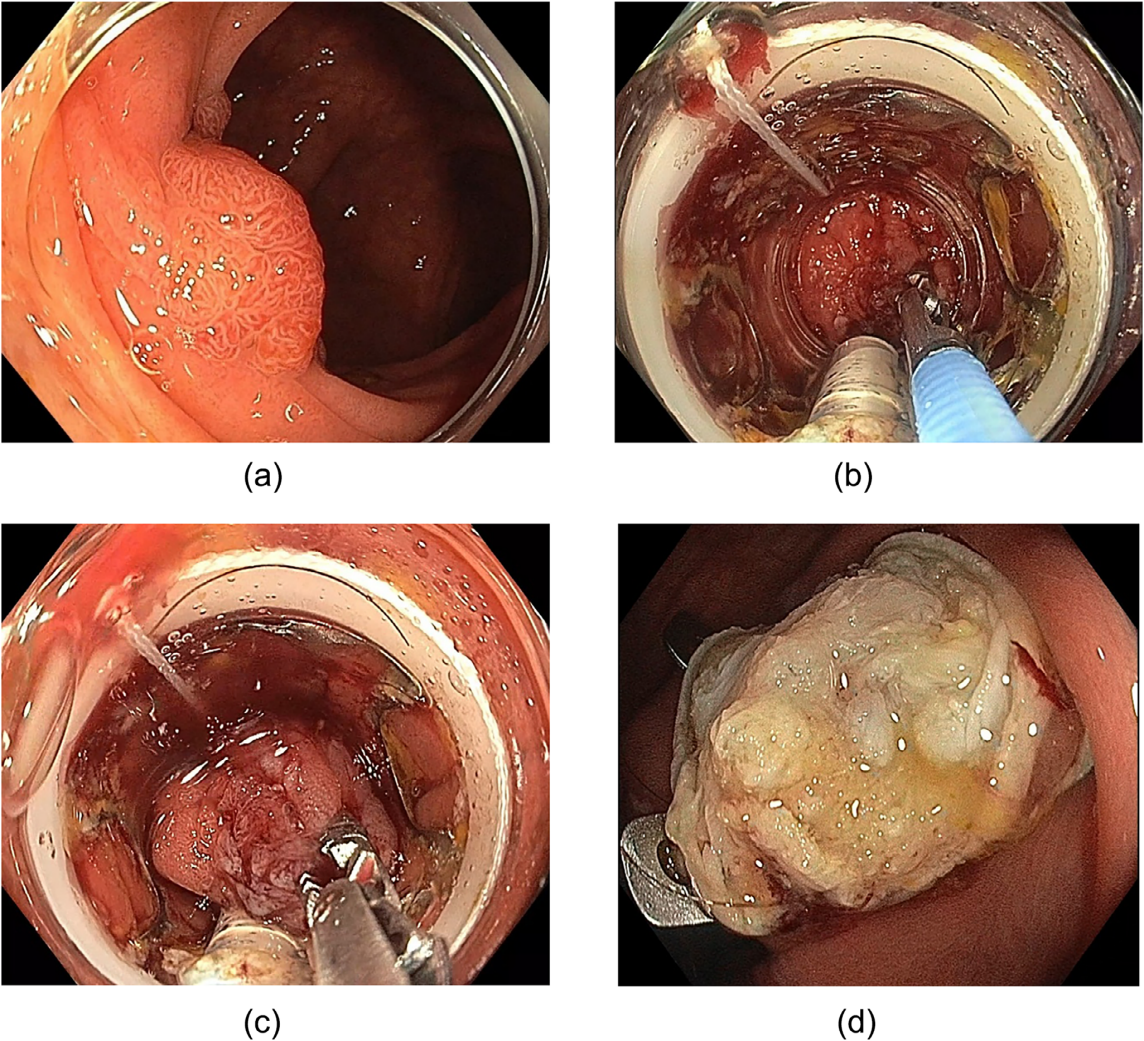
Endoscopic images of colorectal endoscopic full‐thickness resection. (a) White‐light imaging of a non‐lifting adenoma in the ascending colon. (b) Full‐thickness resection device mounted on the distal tip of the colonoscope. (c) Retraction of the target lesion into the device cap using grasping forceps. (d) Resection site following excision with the over‐the‐scope clip in situ.

Advances in endoscopic resection techniques, particularly endoscopic full‐thickness resection (EFTR), have expanded management options for complex colorectal lesions. The full‐thickness resection device (FTRD; Ovesco Endoscopy, Tübingen, Germany) enables en bloc resection of lesions not amenable to standard techniques such as endoscopic mucosal resection (EMR) or endoscopic submucosal dissection (ESD). Increasing evidence also supports its use in selected cases of colorectal cancer with submucosal invasion [1]. FTRD is particularly useful for non‐lifting lesions caused by fibrosis from prior interventions, submucosal invasion, or subepithelial lesions, offering a minimally invasive alternative to technically demanding endoscopic approaches associated with an increased risk of perforation [2] and to surgery, which potentially carries significant morbidity and mortality [3]. EFTR techniques are broadly classified into exposed and nonexposed methods [4]. Exposed EFTR requires advanced ESD expertise, has a steep learning curve, and remains supported by limited evidence for colorectal lesions [5]. In contrast, the FTRD provides a nonexposed approach, whereby a full‐thickness clip is deployed prior to snare resection, enabling immediate defect closure (Figures 1 and 2) [6]. European studies, including a systematic review and meta‐analysis, have reported technical success rates of 84%–90%, R0 resection rates of 78%–82%, and adverse event (AE) rates of 8%–12% [1, 7, 8]. However, data regarding FTRD outcomes outside Europe remain limited, and data regarding predictors of AEs are not defined. This multicenter study aimed to evaluate the technical success, histological outcomes, recurrence rates, and safety profile of colorectal EFTR using FTRD in an Australasian cohort and to identify patient‐ and lesion‐related factors associated with AEs.

## 2 Methods

### 2.1 Patient Selection

This multicenter retrospective cohort study included consecutive adult patients (≥18 years) who underwent colorectal EFTR with FTRD across 13 centers in Australia and New Zealand between January 2018 and July 2025. EFTR with FTRD was applied to lesions <30 mm in the following clinical scenarios when conventional endoscopic resection was not considered feasible: primary non‐lifting lesions; secondary non‐lifting lesions following prior resection (including recurrence or positive margins); lesions involving the appendiceal orifice or arising within a diverticulum; suspected T1 adenocarcinoma; subepithelial lesions; prior endoscopic resection of adenocarcinoma with positive margins; and cases requiring full‐thickness histological assessment for diagnostic purposes. No exclusion criteria were applied. All endoscopists had completed mandatory training through an OVESCO FTRD Accreditation Workshop. Data were extracted from medical records and prospectively maintained databases at each site using a standardized data collection form, with all patient information anonymized. Collected variables included patient demographics, comorbidities, lesion characteristics, procedural details, and histological outcomes. Procedure time was defined as the interval from insertion of the colonoscope with the attached FTRD to scope withdrawal. Technical success was defined as complete macroscopic resection of the target lesion without visible residual tissue. R0 resection was confirmed by histopathological evidence of clear lateral and deep margins. Histopathological findings were classified into a single category per procedure, based on the most advanced histological feature identified within the specimen. Recurrence was defined as histologically proven neoplasia at the EFTR resection site. AEs were categorized as intra‐procedural or delayed, with delayed events defined as occurring >24 h post‐procedure. Minor bleeding was defined as bleeding requiring observation only, whereas major bleeding was defined as bleeding requiring blood transfusion or endoscopic intervention. The primary objective was to evaluate the efficacy (technical success and R0 resection) and safety of colorectal EFTR with FTRD. Secondary objectives included assessment of recurrence and identification of patient‐ and lesion‐related factors associated with AEs.

This study was approved by the Human Research Ethics Committee of the Victorian Translational Research Institute (HREC/106818/Austin‐2024) and conducted in accordance with the ethical standards of the institutional research committee and the principles of the Declaration of Helsinki. Site‐specific authorization was obtained from all participating centers. Given the retrospective design and the use of anonymized data, the requirement for individual informed consent was waived, with all appropriate privacy protections in place.

### 2.2 Statistical Analyses

Continuous variables were summarized as mean ± standard deviation (SD) or median with range or interquartile range (IQR), depending on data distribution assessed using the Shapiro–Wilk test. Categorical variables were expressed as counts and percentages. Comparisons between categorical variables were performed using Pearson's chi‐squared test or Fisher's exact test when expected frequencies were <5. For continuous variables, independent samples *t*‐tests were used for normally distributed data and Mann–Whitney *U* tests for non‐normally distributed data. Univariable logistic regression was performed to evaluate associations between potential risk factors and binary outcomes. Multivariable regression analysis was not performed due to the limited number of outcome events to avoid model overfitting and violation of recommended events‐per‐variable assumptions. Linear regression was applied to evaluate relationships between continuous predictors and outcomes such as procedure time. A two‐sided *p* value <0.05 was considered statistically significant. Statistical analyses were conducted using SPSS version 27 (IBM Corp., Armonk, NY, USA).

## 3 Results

### 3.1 Patient Demographics

A total of 170 colorectal EFTR procedures using FTRD were performed across 13 centers. The mean patient age was 67 years (SD 13.7), and 57% were male. Comorbidities were present in 65% of patients (Table 1). Anticoagulant and antiplatelet agents were withheld for a median of 2 days (range 2–7) before the procedure and resumed after a median of 3 days (range 2–14).

**TABLE 1:** Patient and lesion characteristics, and procedural outcomes.

Patient characteristics	
Age, years (mean ± SD)	67 ± 13.7
Female, *n* (%)	73 (43%)
Comorbidities, *n* (%)	111 (65%)
Antithrombotic use, *n* (%)	50 (29%)
Type of antithrombotic, *n*	
Aspirin, *n*	25
Clopidogrel, *n*	2
DOAC, *n*	21
Warfarin, *n*	4
**Lesion characteristics, *n* (%)**	
Location	
Ileocecal valve	5 (3%)
Appendiceal orifice	41 (24%)
Caecum	10 (6%)
Ascending colon	26 (15%)
Transverse colon	26 (15%)
Descending colon	6 (4%)
Sigmoid colon	19 (11%)
Rectum	37 (22%)
**Histopathological diagnoses, *n* (%)**	
Adenoma	66 (39%)
Low‐grade dysplasia	31
High‐grade dysplasia	20
No dysplasia	15
Sessile serrated lesion	31 (18%)
Low‐grade dysplasia	1
High‐grade dysplasia	1
No dysplasia	29
Lipoma	1 (1%)
Neuroendocrine tumor 5 (3%)	
Normal/benign colonic mucosa	26 (15%)
Adenocarcinoma	
T1	33 (19%)
T2	5 (3%)
T3	1 (1%)
**Procedure outcomes**	
Technical success, *n* (%)	151/170 (89%)
R0 resection, *n* (%)	130/164 (79%)
Recurrence, *n* (%)	11/148 (7.4%)
**Intraprocedural adverse events, *n* (%)**	7/170 (4.1%)
Bleeding	3
Perforation	2
Snare malfunction	2
**Delayed adverse events, *n* (%)**	22/170 (12.9%)
Bleeding, *n*	‐ 11
Appendicitis, *n*	‐ 7
Perforation, *n*	‐ 5
Post‐polypectomy syndrome, *n*	‐ 2

Abbreviations: SD, standard deviation; DOAC, direct oral anticoagulant.

### 3.2 Procedural Characteristics

**FIGURE 3:**
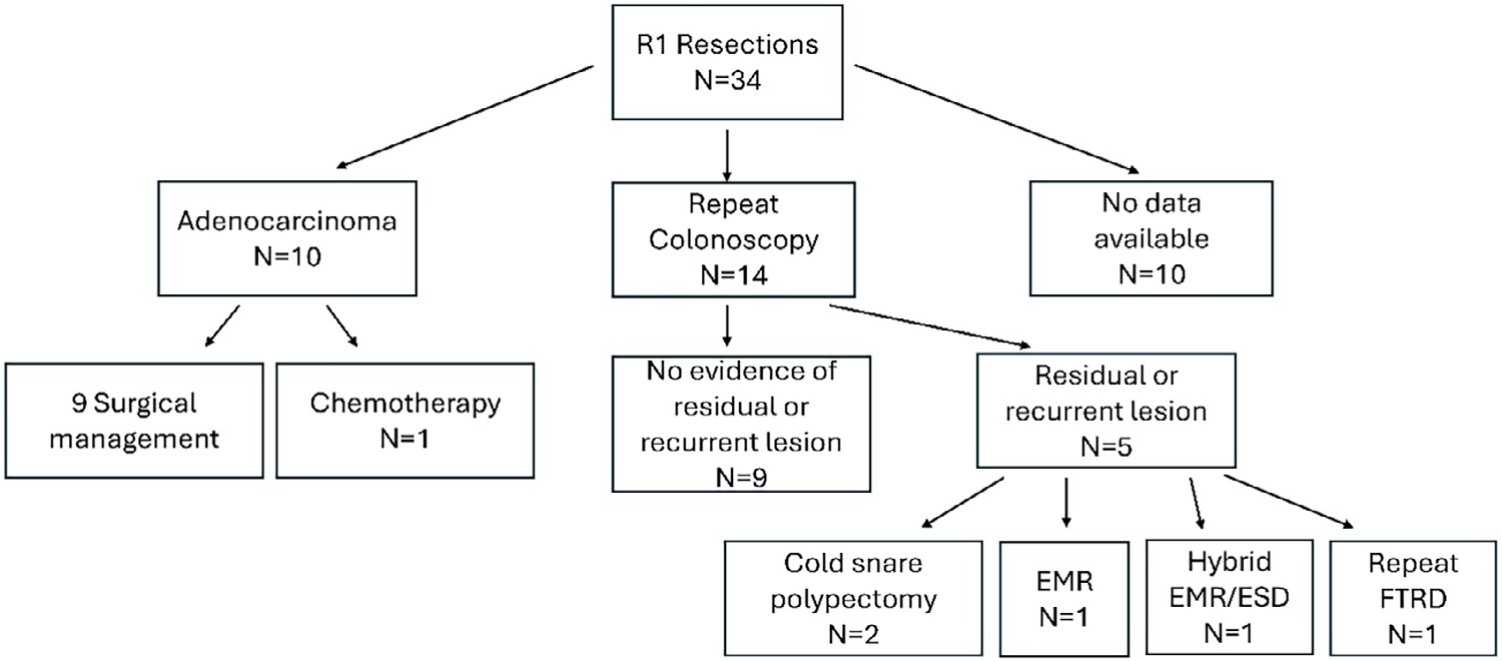
Clinical course following R1 resection. Flow diagram illustrating management strategies and clinical outcomes in patients with R1 resection (histologically incomplete resection/positive margins) after colorectal endoscopic full‐thickness resection. EMR, endoscopic mucosal resection; ESD, endoscopic submucosal dissection; FTRD, full‐thickness resection device.

The mean procedure time was 51 min (SD 28.5), and the mean lesion size was 19 mm (SD 7.3). The appendiceal orifice was the most common resection site (24%). Most procedures (82%) were performed as day cases, and non‐lifting treatment‐naive lesions were the most common indication (Table 2). Technical success was achieved in 89% (151/170) of cases. Histological assessment was available in 168 cases, with two procedures excluded due to failure of resection. For margin analysis, 164 cases were included after exclusion of diagnostic procedures (*n* = 4). R0 resection was achieved in 79% (130/164), whereas R1 resection occurred in 21% (34/164). Further management data were unavailable for 38% (13/34) of R1 cases. Among the remaining 21 cases with follow‐up, 48% (10/21) were adenocarcinomas, all of which underwent additional treatment. Recurrence occurred in 24% (5/21) of followed R1 cases, all of which were successfully managed endoscopically (Figure 3).

**TABLE 2:** Indications for endoscopic full‐thickness resection (EFTR) using full‐thickness resection device (FTRD).

Indications	
Cancer identified after prior EMR/ESD with positive or unclear (Rx) margins	21 (12%)
Rectal EFTR for suspected amyloidosis (diagnostic)	4 (2%)
Appendiceal orifice polyp (%)	39 (23%)
Diverticular‐associated polyp (%)	5 (3%)
Recurrence at prior polypectomy site (not amenable to EMR/ESD) (%)	36 (21%)
Non‐lifting, treatment‐naïve lesion (not amenable to EMR/ESD) (%)	61 (36%)
Subepithelial lesion (%)	4 (2%)

Abbreviations: EMR, endoscopic mucosal resection; ESD, endoscopic submucosal dissection; Rx, resection margins.

### 3.3 Adverse Events

Intra‐procedural AEs occurred in 4.1% (7/170) of cases, including snare malfunction (*n* = 2), bleeding (*n* = 3), and perforation (*n* = 2). One case of intra‐procedural snare malfunction resulted in delayed perforation. Delayed AEs occurred in 12.9% (22/170) of cases, most commonly bleeding (*n* = 11) (Table 1). Bleeding occurred at a median of 3 days post‐procedure. Two patients required endoscopic hemostasis for major bleeding, whereas the remainder were managed conservatively. Delayed AEs were more frequent in appendiceal orifice lesions (29%, 12/41), including delayed perforation in 10% (4/41). Appendicitis occurred in 17% (7/41) of appendiceal cases. Two patients required appendectomy, whereas the remainder were successfully managed with antibiotics (median duration 14 days).

### 3.4 Adenocarcinoma

Histopathology confirmed adenocarcinoma in 39 of 170 lesions (23%). Of these, 12 were well‐differentiated, 21 moderately differentiated, and 6 poorly differentiated. According to the Japanese Classification of Colorectal, Appendiceal, and Anal Carcinoma, 79% (31/39) were classified as high‐risk lesions [9], and 85% (33/39) were pT1 cancers. R0 resection was achieved in 74% (29/39). All 10 R1 adenocarcinomas received additional treatment, including surgery or chemotherapy. Despite achieving R0 resection, seven patients proceeded to surgery due to high‐risk histological features.

### 3.5 Recurrence

Follow‐up data were available for 148 patients and formed the basis of recurrence analysis. The overall recurrence rate was 7.4% (11/148). Several cases were excluded from this analysis, including 16 patients who proceeded to surgery following EFTR (seven due to high‐risk features and nine due to R1 resection), four diagnostic procedures, and two technical failures in which the lesion was not resected. Recurrence was more common following technically unsuccessful procedures (60.0%, 6/10) compared with technically successful resections (3.6%, 5/138). Management of recurrence included cold and hot snare polypectomy, repeat EFTR, and surgical resection. Two patients underwent repeat EFTR using FTRD.

### 3.6 Factors Associated With EFTR With FTRD Outcomes

On univariate analysis, delayed AEs were significantly associated with female sex and right‐sided lesion location. Delayed AEs occurred more frequently in females than males (63.6% vs. 39.9%, *p* = 0.03). Lesions located in the right colon, particularly those involving the appendiceal orifice, were also associated with a higher rate of delayed AEs. Right‐sided lesions were more common in the delayed AE group compared with those without delayed AEs (90.9% vs. 59.5%, *p* = 0.003), and appendiceal involvement was also higher (45.5% vs. 20.9%, *p* = 0.02). Lesion size, procedure time, histology, and use of antithrombotic agents were not significantly associated with delayed AEs.

For delayed bleeding specifically, female sex was again a significant factor (72.7% vs. 40.9%, *p* = 0.04). Larger lesion size was also associated with an increased risk of bleeding (mean 24.6 vs. 18.7 mm, *p* = 0.01). No significant associations were observed between delayed bleeding and antithrombotic use (including aspirin, clopidogrel, direct oral anticoagulants [DOACs], or warfarin), lesion location (right vs. left colon), appendiceal involvement, procedure time, or histology (Table 3).

**TABLE 3:** Univariate analysis of factors associated with overall delayed adverse events and delayed bleeding.

Factor	Delayed AE *n* (%)	No delayed AE *n* (%)	*p* value	Delayed bleeding *n* (%)	No delayed bleeding *n* (%)	*p* value
**Female sex**	14 (63.6)	59 (39.9)	0.03	8 (72.7)	65 (40.9)	0.04
**Antithrombotic use**	7 (31.8)	43 (29.0)	0.48	5 (45.6)	45 (28.3)	0.19
Aspirin	—	—	—	3 (27.3)	22 (13.8)	0.21
Clopidogrel	—	—	—	0 (0)	2 (1.3)	0.87
DOAC	—	—	—	3 (27.3)	18 (11.3)	0.14
Warfarin	—	—	—	0 (0)	4 (2.5)	0.76
**Lesion site**						
Right‐sided	20 (90.9)	88 (59.5)	0.003	9 (81.8)	99 (62.3)	0.16
Appendiceal orifice	10 (45.5)	31 (20.9)	0.02	2 (18.2)	39 (24.5)	0.48
**Lesion size, mm (mean ± SD)**	19.6 ± 8.2	19.0 ± 7.2	0.74	24.6 ± 5.8	18.7 ± 7.2	0.01
**Procedure time, min (mean ± SD)**	58.8 ± 29.3	50.0 ± 28.3	0.23	48.5 ± 22.0	51.3 ± 28.9	0.75
**Adenocarcinoma**	3 (14.3)	34 (27.2)	0.16	2 (18.2)	35 (25.9)	0.44

Abbreviations: AE, adverse events; DOAC, direct oral anticoagulant.

## 4 Discussion

This multicenter cohort study evaluated the efficacy and safety of colorectal EFTR with FTRD in Australia and New Zealand. Our findings demonstrate that colorectal EFTR with FTRD is an effective endoscopic treatment for complex colorectal lesions, with a high technical success rate of 89% and R0 resection rate of 79%, aligning with international data [7, 10, 11, 12]. The overall recurrence rate was 7.4%, with management including endoscopic intervention or surgery.

Technical failure occurred in 19 cases (11%). In two cases, this was due to device malfunction, and in a further two cases, the device could not be advanced to the lesion site because of diverticular narrowing, necessitating surgical management. In the remaining 15 cases, the device was successfully deployed; however, endoscopic assessment demonstrated macroscopically visible residual lesion following resection. Of these, only five developed recurrence. We hypothesize that the residual tissue in the remaining nine cases may have undergone secondary auto‐amputation following clip deployment and subsequent clip detachment. Notably, the deployment technique evolved over the study period. Initially, the clip was deployed prior to snare closure and resection. The current approach involves pre‐closing the snare once the lesion is adequately drawn into the cap to avoid lesion retraction during clip deployment, followed by clip release and subsequent snare resection.

The post‐procedural AE rate was 13%, consistent with previously reported rates of 7%–17% [10, 11, 12, 13]. Delayed bleeding occurred at a median of 3 days post‐procedure (range 1–12 days), similar to timelines observed in EMR and ESD procedures [14, 15]. Nine bleeding cases were managed conservatively, with only two requiring endoscopic intervention. Use of anticoagulants or antiplatelet agents, when appropriately managed, was not associated with an increased risk of delayed bleeding, consistent with findings from EMR and ESD [16, 17, 18, 19, 20]. Resumption of antithrombotic therapy at a median of 3 days post‐procedure did not appear to influence bleeding risk. Our cohort had a mean lesion size of 19 mm, larger than reported in the German registry, suggesting that increased lesion size may be associated with a higher risk of delayed bleeding [7]. Larger lesions often have greater vascularity and require longer healing times due to the extent of tissue damage, leaving the resection site vulnerable until complete healing occurs. Interestingly, female sex may be associated with delayed bleeding. Several anatomical and physiological factors may contribute to this difference. Previous studies have shown that women have longer transverse colons, potentially complicating access and maneuverability during EFTR [21, 22]. Although there is limited evidence to suggest an increased risk of bleeding in females post‐polypectomy [23], a previous study indicated a higher overall AE rate in females, warranting further investigation [24]. Appendiceal lesions pose a significant challenge, often requiring surgery or advanced endoscopic resection [25, 26]. In our cohort, the incidence of post‐FTRD appendicitis was 17%, aligning with reported rates of 14%–17% [27, 28, 29] in the literature. Of the seven patients with appendicitis, only two underwent appendectomy, whereas the remainder were managed successfully with antibiotics. This is lower than the 60%–61% appendectomy rates reported in other studies [27, 29]. Our findings suggest that conservative antibiotic therapy may be effective in selected patients, with an average treatment duration of 14 days, longer than the typical 7–10 days for uncomplicated appendicitis [30]. This raises the question of whether a prolonged course of antibiotics and observation could reduce the need for surgery. The role of prophylactic antibiotics in all patients undergoing FTRD for appendiceal lesions also warrants further investigation.

Repeat EFTR with FTRD occurred in two cases in this cohort, which has not previously been described. The first case involved a 30 mm cecal polyp that had recurred after three prior endoscopic resections. Initial EFTR with FTRD revealed residual polyp, and recurrence was confirmed on follow‐up endoscopy. Repeat EFTR achieved complete (R0) resection. The second case involved a 15 mm transverse colonic polyp with three prior endoscopic resections. Although no lesion was visible after initial EFTR, recurrence was subsequently detected, and repeat EFTR successfully achieved R0 resection. Repeated endoscopic resection attempts increases submucosal fibrosis, making lesion retraction into the FTRD cap technically challenging. Consequently, in recalcitrant or recurrent polyps, early consideration of EFTR with FTRD may improve technical success and reduce the number of interventions required.

Our analysis of colorectal adenocarcinoma cases highlights the emerging role of EFTR in the management of T1b colorectal cancers [1]. Given the small sample size, limited conclusions can be drawn from this cohort. However, evidence supporting the use of EFTR with FTRD for colorectal adenocarcinoma is growing, and this modality should be considered in selected patients, particularly those who are poor surgical candidates. However, the lack of surgical lymph node assessment remains a limitation, as it hinders accurate staging and decisions regarding adjuvant therapy. Further studies are needed to define which subsets of patients with early adenocarcinoma may benefit from EFTR alone and to identify reliable predictors of lymph node involvement.

This study has several strengths. It represents the largest multicenter Australasian real‐world cohort of EFTR, providing insights into practice outside Europe. We conducted a pragmatic evaluation of recurrence patterns and delayed AEs in routine clinical practice. We demonstrated increased recurrence following technical failure, real‐world rates and management of delayed AEs, identification of higher‐risk lesion types (e.g., appendiceal and right‐sided lesions), and successful cases of repeat EFTR. However, several limitations should be acknowledged. Its retrospective design introduces potential selection bias, as only lesions selected for EFTR were included, rather than all lesions referred for endoscopic treatment. The modest sample size may limit generalizability and could underestimate true success and adverse event rates. The observed associations do not account for potential confounding, and the limited sample size precluded multivariable analysis; therefore, findings should be interpreted cautiously. Prospective procedural data were inconsistently available across centers, limiting detailed procedural analysis. Recurrence rates should also be interpreted with caution, as follow‐up colonoscopy was performed in only 45% of patients (66/148). At the primary study center, standard surveillance following R0 resection consists of repeat colonoscopy at 9–12 months. In contrast, for patients with R1 resection who do not proceed to surgery, surveillance is typically undertaken at 3 months. The relatively high attrition rate limits the robustness of recurrence estimates and may reflect the comorbidity burden of the study cohort, potentially reducing suitability for surveillance procedures.

In conclusion, colorectal EFTR with FTRD is a safe and effective minimally invasive technique for managing complex colorectal lesions in the Australia and New Zealand population. Our findings provide further insight into factors associated with AEs and suggest areas for future research, particularly in the prevention and management of delayed complications. Ongoing studies are warranted to refine patient selection, improve procedural safety, and expand the role of EFTR in the treatment of early colorectal cancer.

## Author Contributions

Study conception and design: Sujievvan Chandran and Vicki McGarrigle. Data collection and analysis: Vicki McGarrigle, Yuto Shimamura, Leonardo Zorron Cheng Tao Pu, Erin Horsfall, Anurag Sekra, Alexandra Simpson, Cameron Schauer, Nicholas Wan, Rajvinder Singh, Martin Harb, Milan Bassan, Ben Terkasher, Frank Weilert, Saurabh Gupta, Kwang Chien Yee, Rajan Patel, Ibrahim Hassan, and Byron Theron. Manuscript writing: Vicki McGarrigle, Yuto Shimamura, Sujievvan Chandran, Rhys Vaughan, Marios Efthymiou, Leonardo Zorron Cheng Tao Pu, and Cameron Schauer. Critically reviewed the manuscript: Yuto Shimamura, Sujievvan Chandran, Rhys Vaughan, Marios Efthymiou, Leonardo Zorron Cheng Tao Pu, Cameron Schauer, and Anurag Sekra. All authors read and approved the final manuscript.

## Funding

The authors have nothing to report.

## Ethics Statement

The research received an approval of the Research Protocol by an Institutional Reviewer Board.

## Consent

The authors have nothing to report.

## Conflicts of Interest

Yuto Shimamura is an Associate Editor for Digestive Endoscopy. Other authors declare no conflicts of interest.
